# Cannulated screw and Kirschner fixation for the treatment of medial and lateral malleolar epiphyseal fractures in children: a retrospective study of 36 cases

**DOI:** 10.1186/s13018-019-1287-6

**Published:** 2019-08-08

**Authors:** Linjun Jiang, Jun Wu, Ming Li, Xing Liu, Cong Luo, Xiangyang Qu

**Affiliations:** Department of Orthopaedics, Children’s Hospital of Chongqing Medical University, Ministry of Education Key Laboratory of Child Development and Disorders, Chongqing Engineering Research Center of Stem Cell Therapy, China International Science and Technology Cooperation base of Child Development and Critical disorders, 136# Zhongshan 2 Road, Yuzhong District, Chongqing, 400014 People’s Republic of China

**Keywords:** Epiphysis, Injury, Percutaneous fixation, Minimally invasive, Ankle, Bone union

## Abstract

**Background:**

Percutaneous fixation with cannulated screws is an effective method for treating medial malleolar epiphyseal fractures, which comprise a portion of bimalleolar and trimalleolar fractures. Lateral malleolar fractures also need to be fixed to achieve anatomical reduction and absolute stable fixation of the ankle. However, there are no individual studies in the literature on this topic.

**Methods:**

Thirty-six children (22 boys and 14 girls) aged 8 to 15 years (average, 11.3 years) with medial and lateral epiphyseal fractures were treated by cannulated screw and Kirschner fixation after closed reduction from January 2010 to December 2015 in our hospital. The patients were examined each month postoperatively. Ankle function was assessed using the Baird-Jackson ankle score.

**Results:**

The 36 patients were followed up for 18 to 29 months (average, 25 months). No cases of fracture non-union or secondary displacement were observed, and the healing time was 2.8 ± 1.1 months (range, 2–4 months). At the last follow-up visit, the Baird-Jackson ankle score ranged from 83 to 100 (average, 94), including an “excellent” score in 13 cases, a “good” score in 19 cases, a “fair” score in 4 cases, and a “poor” score in 0 cases. The ankle recovered to the pre-injury level of function within 3.5 ± 1.6 months (range, 2–5 months). Dysfunction, pain, instability, and premature epiphyseal closure were not observed during the follow-up of the 36 patients.

**Conclusions:**

Cannulated screw and Kirschner fixation after closed reduction is an effective and readily available method for the treatment of medial and lateral malleolar epiphyseal fractures in children.

## Background

Malleolar epiphyseal fracture is one of the most common clinical injuries of the ankle joint, accounting for approximately 11% of epiphyseal injuries in children [1–3]. It is considered a “threatening fracture” because the injury mechanism of the distal epiphysis of the tibia and fibula is very complex, and many ankle ligaments are attached at the medial and lateral malleoli. Epiphyseal injuries often cause early arthrosis, joint deformity, and leg length discrepancies [4–6]. Therefore, epiphyseal fractures with more than 2 mm of displacement require surgical treatment to maintain the reduced position of the fracture and/or articular surface, in addition to aiding bone healing [4, 7, 8].

Several types of implants have been used for the anatomical reduction of malleolar epiphyseal fractures, including Kirschner wires, metallic screws, and bioabsorbable screws [4, 9, 10]. Each implant has advantages and disadvantages. Kirschner wires are smooth and cause minimal damage to the epiphysis, but they cannot be used for compression [9]. Therefore, Kirschner wires are typically used to assist in fracture reduction and serve as guides for cannulated screws. Metallic screws and bioabsorbable screws are useful for compression to maintain the reduced position. A retrospective study conducted by Podeszwa et al. indicated that similar results were achieved using metallic screws and bioabsorbable screws in the treatment of malleolar physeal fractures [10]. Bioabsorbable screws eliminate the need for epiphyseal screw removal; however, to date, there have been limited clinical results regarding the application of this method for treating malleolar epiphyseal fractures [11]. Fixation with metallic screws is the most widely used method for treating malleolar epiphyseal fractures.

Percutaneous fixation with cannulated screws is a minimally invasive osteosynthesis technique that offers simple operation and the reliable fixation of malleolar epiphyseal fractures, which has been confirmed in many studies [12–14]. Usually, one or two cannulated screws are placed across the fracture in the medial malleolus parallel to the physeal line [9]. However, medial malleolar epiphyseal fractures comprise only a portion of bimalleolar or trimalleolar fractures [1]. Lateral malleolar fractures also need to be fixed to achieve anatomical reduction and absolutely stable fixation of the ankle [15]. Lateral malleolar fractures are usually fixed with a smooth percutaneous Kirschner wire along the radial axis. However, there are no individual studies in the literature on this topic.

Over a period of 6 years, 36 children with medial and lateral malleolar epiphyseal fractures were treated with cannulated screw and Kirschner fixation after closed reduction in our hospital and were followed up for an average of 2.1 years. In this study, we present their outcomes to evaluate the clinical effects of cannulated screw and Kirschner fixation after closed reduction for the treatment of medial and lateral malleolar epiphyseal fractures in children.

## Methods

### Patients

This study was approved and supervised by the ethics committee of the Children’s Hospital of Chongqing Medical University and was performed in accordance with the ethical standards of the 1964 Declaration of Helsinki. Ninety-eight consecutive cases of medial and lateral epiphyseal fractures were treated from January 2010 to December 2015 in the Children’s Hospital of Chongqing Medical University. Non-displaced malleolar epiphyseal fractures can be managed conservatively. Reduction was performed when the displacement was greater than 2 mm, and open reduction was performed when an adequate anatomical reduction could not be achieved by closed reduction. If the fractures were unstable or at a high risk of secondary displacement, internal fixation was used [4, 7, 8, 16]. Thirty-six of these 98 patients were treated by cannulated screw and Kirschner fixation after closed reduction and were included in the study. The other 62 patients, who were managed conservatively or treated by closed reduction and plaster external fixation or open reduction and internal fixation, were excluded from this study. The guardians of the children provided written informed consent before participation in the study to authorize the publication of the results and the use of photographs of their children.

Patient information is shown in Table 1. The 36 patients (22 boys and 14 girls) were aged 8 to 15 years (average, 11.3 years). The etiology of the fractures was a fall injury in 25 cases and a traffic accident injury in 11 cases. The fractures were categorized according to the Salter-Harris classification system. In this study, all of the medial malleolar epiphyseal fractures were type III, 11 of the 36 lateral malleolar epiphyseal fractures were type I, and the other 25 lateral malleolar epiphyseal fractures were type II.

**Table 1:** Patients’ information (*n* = 36)

Subject	Results
Age (year)	11.3 (8~15)
Sex (*n*)	Boy to girl 22:14
Etiology (*n*)	Fall injury 25
	Traffic injury 11
Side (*n*)	Right to left 14:22
Time to fixation (day)	3.7 (0.5~6)
Number of screws (*n*)	Single to two 23:13
Length of screws (mm)	32~36
Follow-up (month)	25 (18~29)

### Surgical procedure

The surgical procedure was performed according to the Özgür Çiçekli method, which has previously been described in detail [9]. The patients were placed in a supine position, and satisfactory general anesthesia was induced. The affected extremity was draped in a sterile manner. Subsequently, closed reduction was performed. The assistant held the knee of the children against the traction, and the surgeon gripped the distal foot for axial traction. To prevent rotation, the foot was allowed slight plantar flexion under moderate traction intensity. According to the fracture displacement direction and the reverse mechanism of injury, the surgeon pushed the medial or lateral malleolus while pronating or supinating, adducting or abducting, and plantarflexing or dorsiflexing the ankle to reduce the medial and lateral malleolar fractures and achieve a smooth articular surface. After satisfactory reduction was achieved, the medial fractures were temporarily fixed with 1 or 2 guide wires (diameter, 1.0 or 1.5 mm, Treu-Instrumente GmbH, Germany). C-arm X-ray equipment was used to confirm that the guide wire was placed perpendicular to the fracture line and parallel to the physeal line. The cortex was drilled with a cannulated drill in the anterograde direction over the guide wire. Then, cannulated screws (Treu-Instrumente GmbH, Germany) with a diameter of 3 to 5 mm were used for percutaneous fixation. The screw thread should penetrate across the fracture line into the lateral cortex of the distal tibia but should not damage the articular surface or the epiphyseal plate. The lateral malleolar fractures were axially fixed with a smooth percutaneous Kirschner wire (diameter, 1.0 or 1.5 mm, Treu-Instrumente GmbH, Germany) along the radial axis. C-arm X-ray equipment was used to confirm satisfactory fracture reduction and reliable internal fixation.

### Follow-up

All 36 patients were treated with postoperative external fixation of the ankle in the functional position with a plaster cast or brace. The children performed guided flexion and extension exercises of the hip and knee starting from the first postoperative day. The external fixation support was removed after 3–6 weeks (average, 4 weeks), and functional training was started under physician guidance. The patients were followed up every month for the first 3 months, every 3 months for the first year, and then every 6 months for the second and third postoperative years. Walking with full weight bearing was not allowed until the fractures showed bony healing. Ankle function was assessed using the Baird-Jackson ankle score [17]. The maximum possible score was 100, including 15 points for ankle pain, 15 points for ankle stability, 15 points for the ability to walk, 10 points for the ability to run, 15 points for ankle motion, and 25 points for the radiographic results. A total score from 96 to 100, 91 to 95, 81 to 90, and less than 80 was considered excellent, good, fair, and poor, respectively.

## Results

**Fig. 1:**
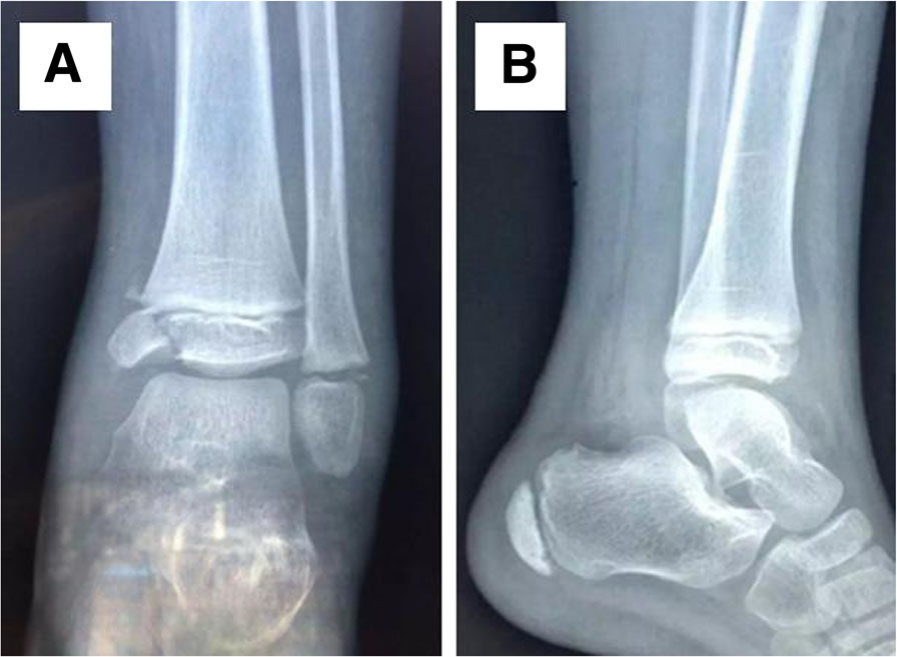
Preoperative X-rays (**a**, the anteroposterior film, **b**, the lateral film) of a 5-year-old boy with medial malleolus epiphyseal fracture (Salter-Harris III) and lateral malleolus epiphyseal fracture (Salter-Harris I) in right ankle joint

**Fig. 2:**
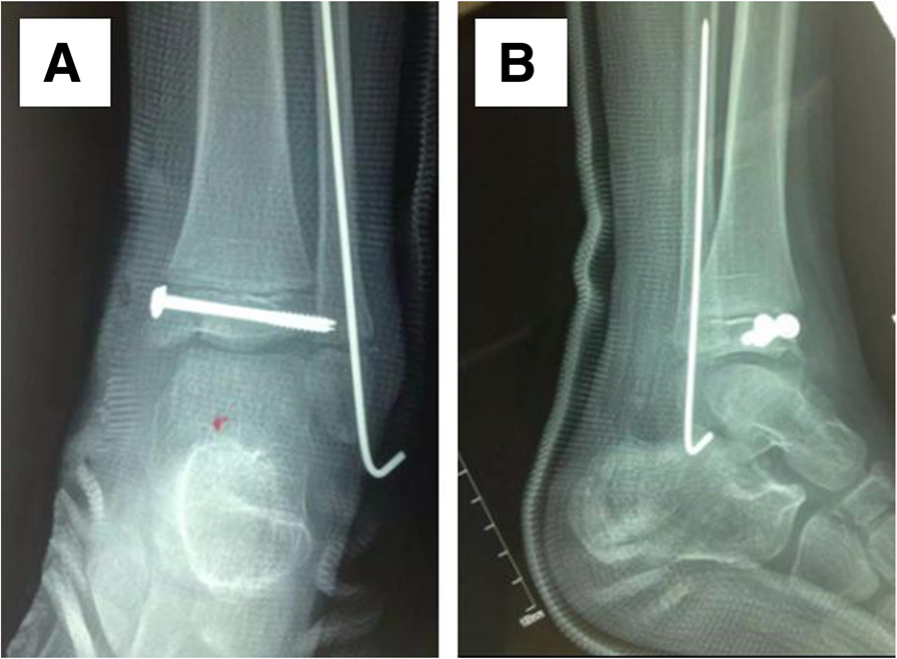
Early postoperative X-rays (**a**, the anteroposterior film, **b**, the lateral film) of the child in Fig. 1. The medial malleolus fracture was fixed with two cannulated screws, and the lateral malleolus fracture was fixed with a Kirschner wire along the radial axis

**Fig. 3:**
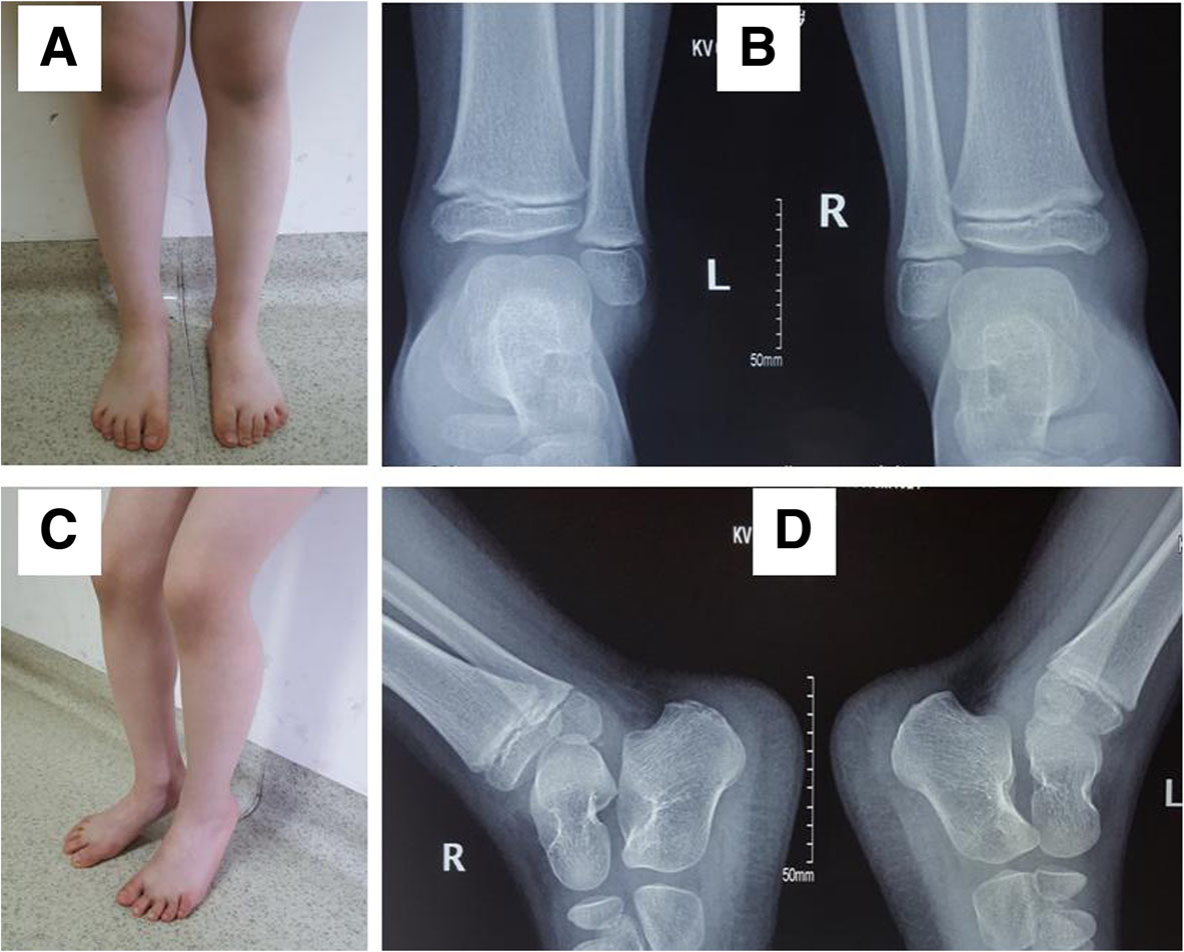
General observation (**a**, **c**) and X-rays (**b**, **d**) of the patient in Fig. 1 at postoperative 2 years indicating that the fracture had healed and ankle joint function had restored

The time from injury to surgical operation ranged from 12 h to 6 days (average, 3.7 days). Twenty-three medial malleolar fractures were treated with a single screw, while the other 13 were treated with two screws. The screws used in this study ranged from 32 to 36 mm in length. The 36 patients were followed up for 18 to 29 months (average, 25 months). Among them, 1 patient experienced numbness of the medial foot, which was caused by local cutaneous nerve stimulation and interference of the implanted screws. The symptom disappeared spontaneously within 2 weeks after the operation. No cases of fracture non-union or secondary displacement were observed. The cannulated screws and Kirschner wires were extracted at 2.8 ± 1.1 months (range, 2–4 months) after surgery when the fractures had healed. At the last follow-up visit, the Baird-Jackson ankle score ranged from 83 to 100 (average, 94). According to the total Baird-Jackson ankle score, the functional outcomes were rated as “excellent” in 13 cases, “good” in 19 cases, “fair” in 4 cases, and “poor” in 0 cases (Fig. 3 and Table 2). The ankle recovered to the pre-injury level of function within 3.5 ± 1.6 months (range, 2–5 months). Premature physeal closure (PPC) or ankle deformities were not observed in any patients at the last follow-up visit. A typical case was shown in Figs. 1, 2 and 3.

**Table 2:** Baird-Jackson ankle score

Criteria	Severity	Score	Cases
Pain	Without pain	15	27
	Mild pain with strenuous activity	12	9
	Mild pain with activities of daily living	8	0
	Pain with weight bearing	4	0
	Pain at rest	0	0
Stability	No clinical instability	15	36
	Instability with sports activities	5	0
	Instability with activities of daily living	0	0
Ability to walk	Able to walk desired distances without limp or pain	15	30
	Able to walk desired distances with slight pain	8	6
	Moderate restriction in ability to walk, with mild pain	6	0
	Able to walk short distances only	3	0
	Unable to walk	0	0
Ability to run	Able to run desired distances without limp or pain	10	12
	Able to run desired distances with slight pain	8	23
	Moderate restriction in ability to run, with mild pain	6	1
	Able to run short distances only	3	0
	Unable to run	0	0
Ability to work	Able to perform usual occupation without restrictions	10	22
	Able to perform usual occupation with restrictions in some strenuous activities	8	13
	Able to perform usual occupation with substantial restrictions	6	1
	Partially disabled, select jobs only	3	0
	Unable to work	0	0
Motion of ankle	Within 10° of uninjured ankle	10	25
	Within 15° of uninjured ankle	7	10
	Within 20° of uninjured ankle	4	1
	< 50% of uninjured ankle	0	0
Radiographic result	Anatomic with intact mortise	25	34
	Mild reactive changes at joint margins	15	2
	Measurable narrowing of superior joint space, with superior joint space > 2 mm	10	0
	Moderate narrowing of superior joint space, with superior joint space 1 to 2 mm	5	0
	Severe narrowing of superior joint space, with joint space < 1 mm, widening of medial clear space, or severe reactive changes (subchondral sclerosis and osteophyte formation)	0	0

## Discussion

The ankle joint is a tongue-like joint with primarily vertical movement consisting of plantar flexion and dorsiflexion in one plane. Only the lateral malleolus can slightly rotate to accommodate variations in the width of the top of the talus. The medial and lateral malleoli are attached by numerous ankle ligaments around the joints. Because of the anatomy and limited range of motion of the ankle joint, the epiphysis of the distal tibia, especially the medial malleolus, often suffers sprain and extrusion. When the foot is fixed, external forces causing varus or valgus movement, plantar flexion, dorsiflexion, or external rotation of the foot can all cause injury to the epiphysis of the distal tibia [18]. In children, the epiphyseal plate is particularly susceptible to injury during the skeletal developmental stages because the epiphyseal plate connection is a mechanically weak zone. Among epiphyseal injuries in children, those to the distal tibia and fibula are considered “threatening fractures” because the injury severity and treatment efficacy directly affect the late functional morphology and development of the ankle [19, 20]. Any damage can cause partially or fully closed epiphyseal plates, resulting in late limb shortening or deformity. According to the Salter-Harris epiphyseal injury classification criteria, the medial malleolar fractures in the included cases involved partial epiphyseal separation with epiphyseal fracture, leading to a joint cavity; these fractures are type III epiphyseal injuries and are considered intra-articular fractures. Fracture displacement can cause articular surface irregularities and mismatch between the ankle mortise and talus because of the role of external forces and stretching of the attached ligaments. The reduced fracture may be unstable and cause late redisplacement, which can in turn result in malunion. Therefore, medial malleolar fractures often require anatomical reduction and effective fixation. The lateral malleolar fractures of the included cases were type I epiphyseal injuries with epiphyseal separation. These fractures generally do not involve damage to the articular surface and cause relatively minor displacement compared to medial malleolar fractures. The cases included in this study included medial malleolar fractures with significant displacement involving the articular surface, accompanied by lateral malleolar fractures with relatively minor displacement and no involvement of the articular surface. These findings are consistent with the injury mechanism, the characteristics of the ankle joint anatomy, and the characteristics of medial and lateral malleolar epiphyseal fractures.

The selection of a treatment for medial and lateral malleolar epiphyseal fractures is very important for functional ankle recovery in children. The epiphysis is not only the point of bone growth in children but also a weak area prone to injury under external forces. A personalized and selective approach for the treatment of medial and lateral malleolar fractures should be applied based on various factors, such as the degree of fracture displacement and the severity of accompanying local injury to soft tissue. Non-displaced malleolar epiphyseal fractures can be managed conservatively, and the tube or “U”-shaped plaster fixation needs to be replaced after the swelling has subsided. Reduction was performed when the displacement was greater than 2 mm, and open reduction was performed when an adequate anatomical reduction could not be achieved with closed reduction. If the fractures were unstable or at a high risk of secondary displacement, internal fixation was adopted. Moreover, open reduction and internal fixation were performed when the fractures were accompanied by open wounds, ankle dislocation, or severe ligament injury or in fractures associated with blood vessel and nerve injury [4, 7, 8, 16, 21]. In the present study, medial and lateral malleolar epiphyseal fractures with displacements of more than 2 mm were treated by cannulated screw and Kirschner fixation after closed reduction. Closed reduction and internal fixation can effectively reduce and fix fractures, does not cause additional injury to the local soft tissue and periosteum, and only slightly interferes with fracture healing. Therefore, it shortens the healing time and allows the early recovery of limb function [22].

The most common and important complication of distal tibia physeal fracture is PPC [21]. As shown in Table 3, the incidence of PPC ranges from 0.5 to 43% [2, 9, 16, 21, 23–26]. The factors that contribute to the development of PPC are still controversial. Özgür Çiçekli et al., Russo et al., and Leary et al. found that PPC was significantly associated with the mechanism of injury [9, 23, 24], while Seel et al. found no significant link between the mechanism of injury and PPC [25]. Leary et al. demonstrated that the primary fracture displacement had a significant influence on PPC [24], but this was disapproved by other studies [2, 23, 25]. Many studies have confirmed that fibula fracture is an important risk factor for the development of PPC, which furthermore indicates that good anatomical reduction may reduce the rate of PPC [2, 16, 25, 26]. In this study, medial malleolar fractures were treated by cannulated screws after closed reduction, and lateral malleolar fractures were effectively fixed by the axial implantation of a smooth Kirschner wire. PPC or ankle deformities did not occur in this study. Because this study is limited by its retrospective nature, and only children treated by cannulated screw and Kirschner fixation after closed reduction were included, a prospective randomized controlled study should be performed to confirm the clinical efficacy and investigate the factors associated with complications.

**Table 3:** The studies on pediatric epiphyseal ankle fractures and premature physeal closure (PPC)

Study	Number of patients (*n*)	Mean age (years)	Follow-up (years)	PPC (*n*)	Factors associated with PPC	Factors do not induce PPC	Malformation (*n*)
Russo et al. [23]	96	12.6	Mean 0.6	40	Mechanism of injury	Primary fracture displacement Associated distal fibular fracture	1
Özgür Çiçekli et al. [9]	24	12.3	Mean 1.1	3	Mechanism of injury		0
Leary et al. [24]	124	12.5	Mean 1.1	15	Mechanism of injury Primary fracture displacement		5
Seel et al. [25]	225	12.5	Mean 5.7	12	Associated distal fibular fracture Good anatomical reduction	Mechanism of injury Primary fracture displacement Residual displacement after reduction Treatment modality	6
Cai et al. [2]	286	11.7	Mean 6.4	42	Associated distal fibular fracture	Primary fracture displacement Residual displacement after reduction	16
D’Angelo et al. [16]	46	11	More than 2	1	Associated distal fibular fracture Good anatomical reduction		1
Schurz et al. [26]	195	11.6	Mean 0.2	1	Good anatomical reduction		1

## Conclusions

In this study, cannulated screw and Kirschner fixation after closed reduction was used to treat medial and lateral malleolar epiphyseal fractures in children, and the results confirmed a high rate of union and satisfactory functional outcomes without complications. Hence, cannulated screw and Kirschner fixation after closed reduction is an effective and readily available method for treating medial and lateral malleolar epiphyseal fractures in children.

## Data Availability

The datasets analyzed in the study are available from the corresponding author on reasonable request.
